# Comparison of bedside ultrasonography and bedside chest radiography in neonatal peripherally inserted central catheters: A before and after self-control study

**DOI:** 10.3389/fped.2022.976826

**Published:** 2022-10-18

**Authors:** Xuexiu Liu, Xiaojun Tao, Ye Xu, Xianhong Zhang, Yanhan Chen, Liping Wu

**Affiliations:** ^1^ Department of Neonatology, Children's Hospital of Chongqing Medical University, Chongqing, China; ^2^ Radiology Department, China International Science and Technology Cooperation Base of Child Development and Critical Disorders, Chongqing Key Laboratory of Pediatrics, Chongqing, China; ^3^ College of Nursing, Chongqing Medical University, Chongqing, China; ^4^ Department of Nursing, Children's Hospital of Chongqing Medical University, Chongqing, China

**Keywords:** peripherally inserted central catheter, ultrasonography, chest radiography, positioning, neonate

## Abstract

**Objective:**

This study aimed to compare the applications of bedside ultrasonography (US) and bedside chest radiography (CR) in positioning peripherally inserted central venous catheter (PICC) at Neonatal Intensive Care Units (NICUs).

**Methods:**

The study is a prospective before and after self-control clinical trial. A consecutive series of 181 neonate patients were finally enrolled for PICC placement. CR, followed by US, was used to evaluate and readjust the sites of catheter tips. The imaging capability for PICC key structures, fluctuation of the measured data, measurement of tip-to-atrium distance, operation time, infants' body temperature changes, and direct expenses of the two imaging modalities were obtained and compared.

**Results:**

(1) Comparison in viewing PICC key structures: the display rate of catheter tip, SVC-and-right-atrium junction, IVC-and-right-atrium junction and tip-to-atrium distance is 99.47%, 100%, 100% and 99.47% for US and 100%, 98.42%, 97.37% and 95.79% for CR, respectively. (2) Fluctuation of the measured data by US and CR: the tip-to-atrium distance measured by US is 0.631 (0.435–0.820) cm, and that measured by CR is 0.593 (0.210–0.825) cm. US showed a narrower range of datum variance. (3) Consistency between US and CR: for consistency analysis, the Kappa coefficient (*κ*) was 0.843 (*P* < 0.05), showing their favorable consistency. (4) Comparison of operation time and infants' body temperature drop: for a CR exam, the time period taken was significantly longer than that of US (59.7 ± 26.33 vs. 79.6 ± 28.06, *P* < 0.001); and CR operations caused a significant babies' body temperature drop compared to US (0.14 ± 0.11 vs. 0.34 ± 0.19, *P* < 0.001). (5) Comparison of the direct expenses: the total cost for CR positioning was significantly higher than that for US (¥153.99 vs. ¥143, *P* = 0.026).

**Conclusion:**

US exhibited superior traits to CR in the positioning of PICC tip. It could be promising for routine use in NICU.

## Introduction

Peripherally inserted central catheter (PICC) is a technique by inserting a catheter through peripheral veins so that the catheter tip is placed in the superior vena cava (SVC) or inferior vena cava (IVC) to establish a safe and stable infusion pathway. Currently, PICC has been widely used in Neonatal Intensive Care Units (NICU) for intravenous nutritional support and long-term drug infusion. Ensuring the catheter tip within the vena cava is critical because malposition may induce adverse outcomes such as infectious endocarditis, atrial fibrillation, pleural effusion, etc (1, 2). Clinically, chest radiography (CR) has been applied as the “gold standard” to confirm the sites of catheter tips (3, 4). However, accumulating evidence showed the drawbacks of CR positioning including complex operation requirements, nondynamic and retrospective imaging, ionizing radiation, longer time consumption, etc (3). All current guidelines recommend avoiding x-ray on neonates as much as possible; x-ray is unsafe in neonates, and radiation exposure may increase the risk of cancer or other diseases (5). Compared to CR, ultrasonography is easier to operate at bedside and able to view catheter tips and cardiovascular structures in real-time without ionizing radiation. US-based tip location can be repeated safely and without significant cost, every 24 h or every 48 h, or whenever needed, to check the tip of the PICC is still in the correct position because malposition of the tip would be logistically sustainable and unsafe for the child.

Ultrasound (US) devices are becoming increasingly available in many neonatal intensive care units as a tool the teams can use in routine clinical care (6). Largely because of the many advantages, it has recently been used, with success, for the evaluation of PICC location in adult patients (7). US for catheter placement is not the current standard of practice, however, because of limitations of cost of equipment and the perceived high degree of training required to perform US routinely for catheter placement. And, due to the lightweight and small blood vessels of newborns, it is difficult to accurately find the position of the catheter tip, which greatly increases the difficulty and accuracy of neonatal PICC positioning (8). At present, PICCs are inserted and advanced blindly to a predetermined length based on an external anatomic measurement of the estimated catheter pathway. In order to check the adequacy of catheter placement, CR was placed after catheter placement. Frequently, these catheters are not placed at an optimal position the first-time necessitating repositioning the catheters followed by further CR. This involves the movement of often critically ill infants, extending time away from optimal nursing care, as well as radiation exposure (9). During repositioning before permanently fixing the catheter, the catheter may shift, which is also a significant risk (10). Therefore, we hope to find a more suitable PICC localization method for newborns. Whether US localization of PICC can overcome the problem of CR localization in newborns and whether it will be more suitable for newborns needs further research. To our best knowledge, only a few studies were reported about its application in NICU.

Hence in this study, we enrolled a consecutive series of 181 neonates, aiming to understand more about the values of bedside US in neonatal PICC locating, by comparison with bedside CR.

## Methods

### Estimation of sample size

The sample size was estimated by using “confidence intervals for kappa” of PASS 15.0 software. The main purpose of this study is to compare the applications of US and CR in positioning PICC at NICUs. We study the consistency of the two methods. Because of the counting property, we use the Kappa index to evaluate the data. When setting the parameters, a kappa coefficient between US and CR was estimated to be about 0.843, and the standard deviation was 0.12. If the class I error of the relevant parameters is set as 0.05 (*α* = 0.05) with statistical efficiency of 0.95, and the calculated sample size was 89. With an addition of 10% sample loss, at least 98 patients are required.

### Participants

A consecutive series of 190 neonates who needed PICC in NICU of Children's Hospital of Chongqing Medical University were enrolled in the study. The period of recruitment was from April 2021 to August 2021. Inclusion criteria are newborns: (1) asking for PICC; (2) able to tolerate US and CR exams; (3) with normal coagulation time; (4) without severe contraction or collapse of peripheral blood vessels; (5) having informed and signed informed consent by the parents. Exclusion criteria are newborns: (1) with cardiovascular diseases; (2) with spinal deformity; (3) experiencing failed PICC attempts; (4) having contraindications of PICC including infection, skin allergy, skin injury, and phlebitis, immune deficiency disease, abnormal bleeding and coagulation time and severe collapse of peripheral blood vessels, etc. The institutional review board has approved the study (Approval No., 2021-159; clinical trial registration No., ChiCTR2100045948).

### Placement of PICC line

PICC placement was performed by two nurses with PICC operation qualifications. Briefly, the child was placed in an incubator. Catheterization was performed with a puncturing kit containing 26 GA (1.9 F) single-lumen PICC catheters according to the neonatal PICC catheterization operation specifications (11). After inserting the line, CR was taken to locate the catheter tip, then followed with US for relocating and guided adjustment.

According to the specifications, it should be avoided placing the catheter tip in the heart of neonates and infants (12). The optimal tip position complied with the recommendation of the 2016 guidelines by the American Infusion Nurses Society (INS), i.e., the safest PICC tip be located within the lower third of SVC or just below the IVC-and-right-atrial junction (13–15).

### Locating catheter tips by CR

CR was conducted under a 0.7/1.3U163C-36 system (Shimadzu, Japan). An experienced radiologist and a PICC specialist nurse read the images together. According to the INS guidelines, a catheter tip at the level of 4th–6th thoracic vertebrae is regarded as the optimal placement for upper limb PICC and at the level of 8th–10th thoracic vertebrae for lower limb PICC (13). Besides, our hospital also took the tracheal carina and the right cardiophrenic angle as the imaging marks of the SVC-and-right-atrium junction and IVC-and-right-atrium junction, respectively.

### Locating catheter tips by US

**Figure 1:**
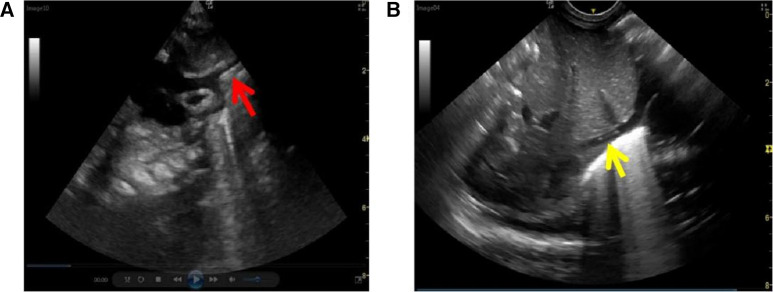
PICC ultrasonic imaging of vena cava. (**A**) superior vena cava (red arrow), and (**B**) inferior vena cava (yellow arrow).

US is conducted under a LOGIQ e color Doppler ultrasonic diagnostic system (6S and 8C probes, GE Company, USA) by two nurses who also executed PICC placement. Both of them have taken training and acquired an ultrasonic operation qualification certificate and had at least 3 years of clinical experience in US. The ultrasonic probe was set at the midline of the subxiphoid region or the parasternal line of the right subclavicle region. A hyperechoic “equal sign” like or sandwich-like structure would be detected within the vena cava, which represents the inserted line. In detail, for clearly viewing the “equal sign” like echoes of the catheter tip in SVC, the probe was placed longitudinally at the 2nd–3rd intercostal spaces on the right of the sternum to delineate the long axis of the aortic arch and the short axis of SVC, and then rotated clockwise for about 15° and tilted slightly to the right to show the long axis of SVC and the right atrial entrances of SVC and IVC (Figure 1A). Under US guidance a small dose of 0.9% sodium chloride solution was injected into the catheter to confirm the position of the tip. Subsequently, the distance between the tip and the right atrial inlet (thereafter referred to as “tip-to-atrium distance”) was measured and the improper tip position was US-guided readjusted. For clearly viewing the “equal sign” like echoes of the catheter tip in IVC, the probe should be placed longitudinally at the midsagittal position of the subxiphoid region and scan along the inferior rib to delineate the IVC and right atrial inlet (Figure 1B). The tip-to-atrium distance was measured and the improper tip position was readjusted.

### Observation and analysis

#### Comparing US with CR in visualizing the key structures at PICC

Catheter tip, right-atrium inlets of SVC and IVC, and tip-to-atrium distance that can be viewed on US and CR was recorded for each patient. US-visible right-atrium inlets of SUV or IVC are defined when it visually depicts the superior or inferior inlet of the right atrium. CR-visible right-atrium inlets of SUV or IVC are defined when it clearly images the tracheal carina or the right cardiophrenic angle. Measurement of tip-to-atrium distance is considered feasible when both of the tip and the right-atrium inlet are shown on a single image of US or CR.

#### Fluctuation of the measured data by US and CR

Tip-to-atrium distance of each patient was measured separately by US and CR. The quartile is calculated to represent fluctuation of the measured data, and fluctuation between the two data sets was compared. Narrower data fluctuation indicates the measurements made by the imaging tool are more stable and reliable.

#### Consistency between US and CR

To further clarify the validity of US, tip-to-atrium distances measured by US and CR were compared to investigate their consistency and correlation (16). For the sake of calculation convenience, both of the data sets of US and CR were transformed into three scores that were 1, 2, and 3, respectively.

#### Comparison of operation time and babies' body temperature drop between US and CR

The time period for each imaging checkup was recorded in minutes using a stopwatch. The time period recorded was defined as from the start of the imaging procedure to the time point the operators had confirmed the tip position. Data of the time periods were compared between US and CR. In addition, babies' body temperature drop brought by US exam and CR exam was compared as well. For a bedside CR exam, babies must be taken out of incubators and sent to a dedicated room in NICU. The process may lead to babies' body temperature drop.

#### Comparison of the direct expenses between US and CR

The direct cost of US and CR in locating the tips were recorded and compared (17).

#### Evaluation of catheterization results

Blind methods were applied to enhance the reliability of the results: (1) both the two operators of US and CR do not know the purpose and significance of this study; (2) a third person was asked for the data collection and statistical treatment, who also did not know the study purpose and the equipment producing the data.

### Statistics

The software SPSS 24.0 was used to statistically process the data. Data with normal distribution were expressed in mean ± standard deviation (*x* ± *s*); the count data were expressed in the number of cases and percentage (%), and fluctuation of data was expressed in quartile. Intra-group correlation coefficient (ICC), and Kappa coefficient analysis was used to investigate consistency between US and CR. Paired *t*-test was used to test the inter-group difference in operation time, body temperature change, and direct cost. *P* < 0.05 means the difference is statistically significant.

## Results

### Patients

One hundred and ninety babies were initially enrolled in this study. Except nine cases were excluded for failed PICC attempts, the remaining 181 babies, 90 males, and 91 females, were finally in the cohort, including 64 of upper limb placement and 117 of lower limb placement. Their average gestational age and average birth weight were (31.74 ± 2.58) weeks and (1630.51 ± 529.36) g, respectively.

### Comparing US with CR in visualizing the key structures at PICC

The comparison between US and CR in viewing the catheter tips and key anatomical structures was summarized in Table 1. US failed to show the position of the PICC tip in only one case, whereas CR failed to show the position of SVC-and-right-atrium junction in three babies and the position of IVC-and-right-atrium junction in five.

**Table 1:** Comparison of the imaging capability for the key structures between US and CR at PICC.

	PICC tip	SVC-and-right-atrium junction	IVC-and-right-atrium junction	Tip-to-atrium distance
Cases viewed by US (%)	189 (99.47)	190 (100)	190 (100)	189 (99.47)
Cases viewed by CR (%)	190 (100)	187 (98.42)	185 (97.37)	182 (95.79)

### Fluctuation of the measured data by US and CR

The tip-to-atrium distance measured by US is 0.631 (0.435–0.820) cm, and that measured by CR is 0.593 (0.210–0.825) cm. The dispersion degree of the measured tip-to-atrium distances represents their fluctuation. Compared to CR, US showed a narrower range of datum variance, indicating its favorable stability and reliability in measurement.

### Consistency between US and CR

The tip-to-atrium-distance measurements of CR presented a skewed distribution, which we think is largely due to the inappropriate patients' positions at image acquisition. For the sake of calculation convenience, both of the data sets of US and CR were transformed into three scores, i.e., a distance less than 0 cm was scored as 1; 0–1 cm was scored as 2; greater than 1 cm was scored as 3. Scores 1, 2, and 3 separately represented PICC failure, satisfaction, and fairness; and scores 2 and 3 are viewed as “PICC success.” The scores were compared between US and CR. Resultantly, for consistency analysis, the Kappa coefficient (*κ*) was 0.843 (*P* < 0.05), showing their favorable consistency. Notably, in two patients satisfactory PICC tip position was shown on CR, but a followed US demonstrated the tips go wrongly into cardiac cavities. The tips were subsequently withdrawn into SVC under US guidance.

### Comparing operation time and babies' body temperature drop between US and CR

**Figure 2:**
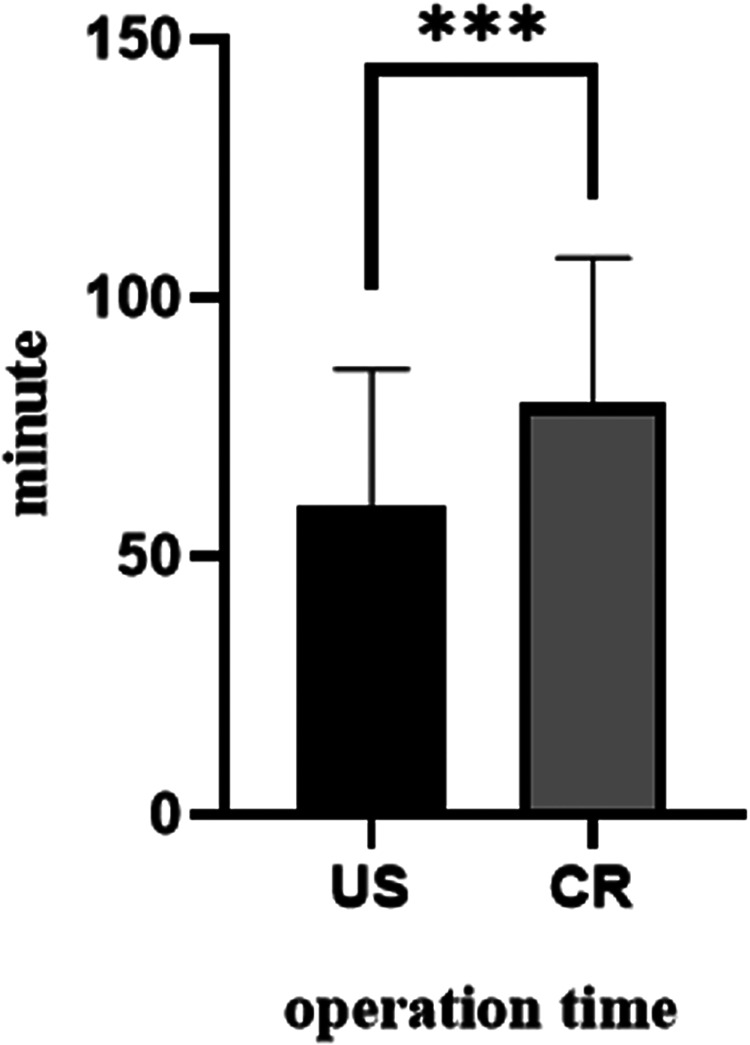
Comparison of operation time between US and CR (***P < 0.001).

**Figure 3:**
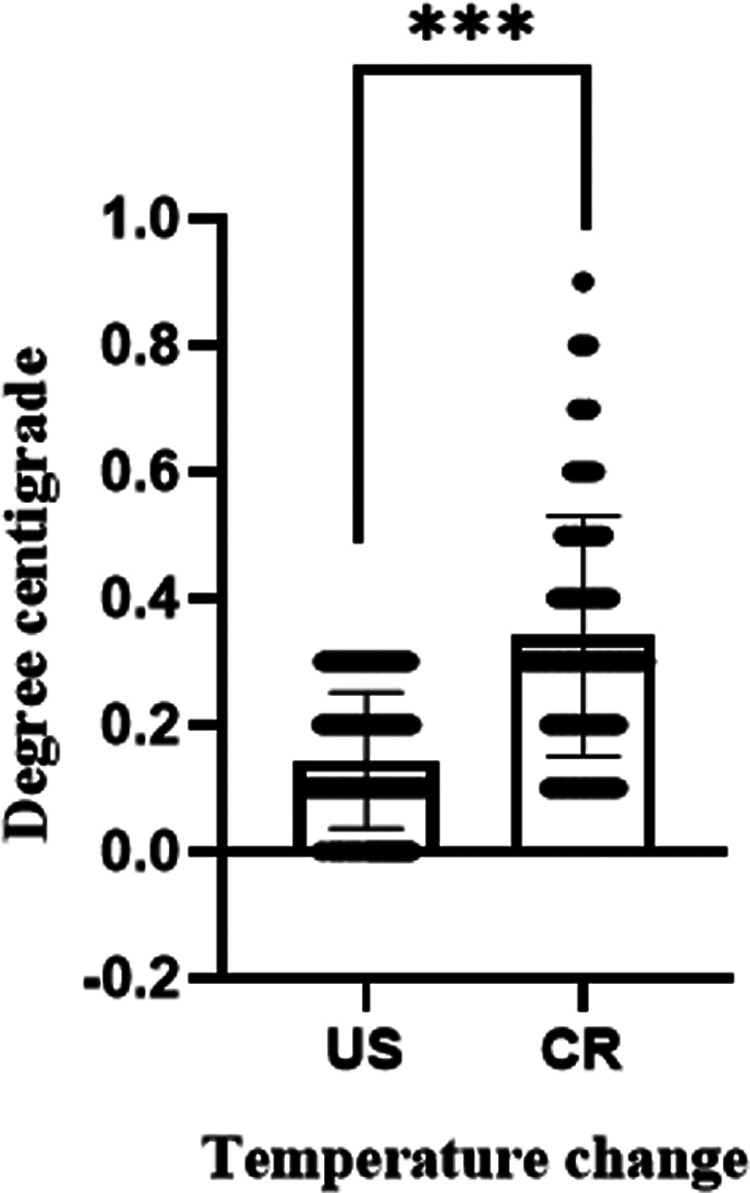
Comparison of temperature change between US and CR (***P < 0.001).

For a CR exam, the time period taken was significantly longer than that of US (59.7 ± 26.33 vs. 79.6 ± 28.06, *P* < 0.001, Figure 2). Moreover, to have a CR exam, infants must be taken out of their incubators and sent to a special room. Such CR operations caused a significant babies' body temperature drop compared to US (0.14 ± 0.11 vs. 0.34 ± 0.19, *P* < 0.001, Figure 3).

### Comparing the direct expenses between US and CR

**Figure 4:**
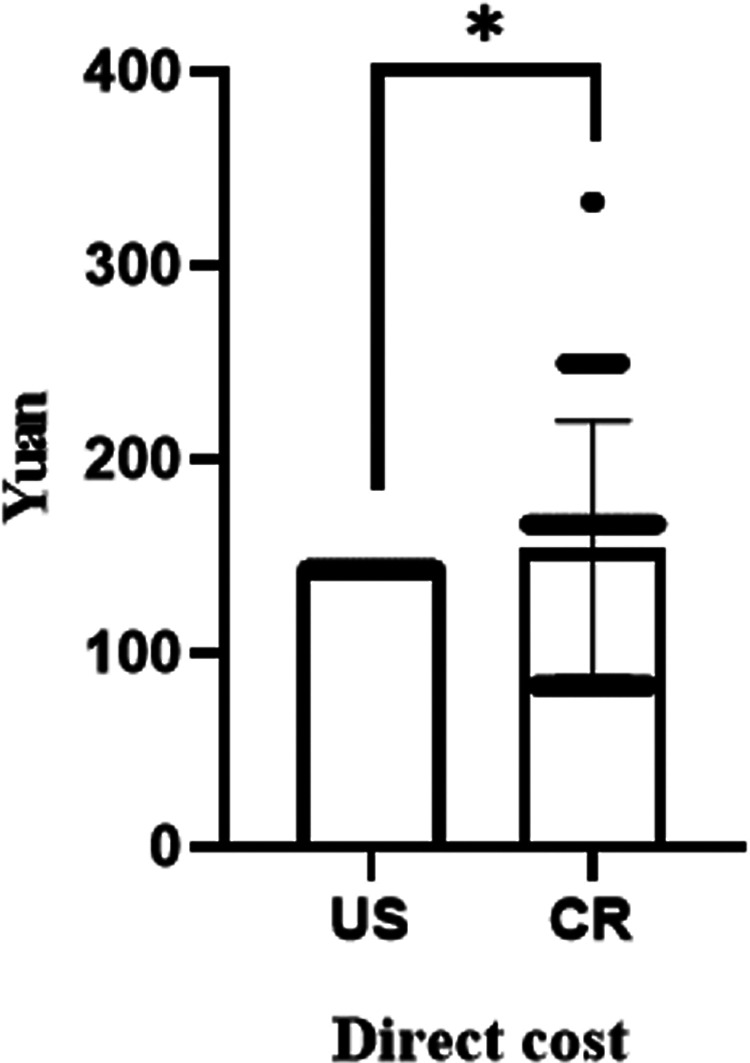
Comparison of direct cost between US and CR (*P = 0.026).

Positioning of PICC by US and CR would separately be paid ¥153.99 and ¥143 on average. The total cost for CR positioning was significantly higher than that for US (*P* = 0.026, Figure 4).

## Discussion

Traditionally, CR was regarded as the “gold standard” for PICC positioning. But current guidelines recommend that the final location of the tip should be assessed during the procedure itself (11). This is possible with US, since the clinician can assess the position before the final securement of the PICC, but it is not possible with CR, since the clinician has to secure temporarily the PICC, perform the x-ray and then secure the PICC in a definite way. Just this consideration alone should be enough to support the use of US as the golden standard for tip location of PICCs in neonates.

US is also extremely useful to direct properly the catheter during the tip navigation, which is impossible with x-ray unless adopting fluoroscopy (18). However, the position of the PICC tip on a CR picture is readily affected by improper patient position, blurred bronchial carina, and thoracic malformation. x-ray based tip location is inaccurate since it is just a statistical guess based on radiological landmarks. The clinician does not “see” the cavo-atrial junction, but takes a chance based on the statistical relationship between the cavo-atrial junction and the radiological landmark. On the contrary, US actually “sees” the catheter and the vasculature, including the cavo-atrial junction. It is not a statistical estimate, but a real visualization of the position of the tip (13). At US imaging, the catheter in the blood vessel presents a special image structure of “high and low,” just like the equal sign “=.” SVC, IVC, and their right-atrium inlets can also be depicted clearly by US. These merits provide grounds for US-guided PICC placement (19). In fact, US has been used in adult patients to guide and adjust PICC placement and has been demonstrated largely reducing the placement-and-positioning related complications (20). In this study, we focused on its applications in newborns in NICU.

Different from the previous studies, this study adopted a prospective before and after self-control method in the hope of obtaining more comparable measurements between US and CR (21). The results showed that US and CR have significant consistency in locating the tip position, as was also confirmed by other studies (22). We then compared the stability of data measured by US and by CR. Resultantly, the data of US presented definitely narrower fluctuation than those of CR, suggesting its nice reliability. Notably, we do not think the wide fluctuation of CR data results only from unsatisfied placement but tend to think it from inaccurate measurements caused by the effects aforementioned. So herein we did not think it is appropriate to take CR as the “gold standard” method at PICC positioning. This is why we did not test the specificity and sensitivity of US positioning. As for the operation time, US exhibited less time consumption than that of CR (*P* < 0.05). Less operation time means reduced risk of complications (16). Measurement of the infants' body temperature change revealed that positioning by CR incurred a significant body temperature drop than by US. Complex preparation, more time for exams, and operating out of incubators mean that the infants would expose to the indoor air for a longer time. A long time out of the incubator would induce hyperthermia in the infants (23–26). Different from CR, US exam can be conducted totally within incubators. Besides, we recorded both the cost for US and CR and found that a direct expense for US positioning was ¥143 on average, significantly smaller than ¥153.99 for CR positioning.

### Limitations

This study has at least three limitations. First, due to the wide fluctuation of CR measurements, this study did not calculate the specificity and sensitivity of US positioning. Second, we did not execute a longitudinal study to in-depth understand the pros and cons of US in PICC placement. Third, only a univariate comparison was used for the study.

## Conclusions

In general, this study showed that US positioning of PICC tip exhibited statistically significant advantages to CR, including visual evaluation of catheter tip and the cardiovascular system, real-time and dynamic imaging, no x-ray exposure, relatively less operation time, and low cost. These advantages may bring a high success rate and safety at neonates' PICC placement. Thus, we conclude that US, although currently not the standard of practice, could be promising for routinely use in the neonates' PICC placement at NICU.

## Data Availability

The original contributions presented in the study are included in the article/Supplementary Material, further inquiries can be directed to the corresponding author/s.

## References

[1] Kolaček S Puntis JWL Hojsak I. ESPGHAN/ESPEN/ESPR/CSPEN guidelines on pediatric parenteral nutrition: venous access. Clin Nutr. (2018) 37:2379–91. 10.1016/j.clnu.2018.06.952 30055869

[2] Weil BR Ladd AP Yoder K. Pericardial effusion and cardiac tamponade associated with central venous catheters in children: an uncommon but serious and treatable condition. J Pediatr Surg. (2010) 45:1687–92. 10.1016/j.jpedsurg.2009.11.006 20713221

[3] Han Z Liu G Zhang H. Advances in the application of peripheral central venous catheter tip positioning technology. Zhongguo Yi Liao Qi Xie Za Zhi. (2020) 44:56–9. 10.3969/j.issn.1671-7104.2020.01.012 32343068

[4] S N. Are supine chest and abdominal radiographs the best way to confirm PICC placement in neonates? Neonatal Network. (2010) 29:23–35. 10.1891/0730-0832.29.1.23 20085874

[5] Battiwalla M Fakhrejahani F Jain NA Klotz JK Pophali PA Draper D Radiation exposure from diagnostic procedures following allogeneic stem cell transplantation – how much is acceptable? Hematology. (2014) 19:275–9. 10.1179/1607845413Y.0000000131 24094072 PMC4155497

[6] Jain A McNamara PJ Ng E El-Khuffash A. The use of targeted neonatal echocardiography to confirm placement of peripherally inserted central catheters in neonates. Am J Perinatol. (2012) 29:101–6. 10.1055/s-0031-1295649 22105438

[7] Subramanian S Moe DC Vo JN. Ultrasound-guided tunneled lower extremity peripherally inserted central catheter placement in infants. J Vasc Interv Radiol. (2013) 24:1910–3. 10.1016/j.jvir.2013.08.020 24267528

[8] Tauzin L Sigur N Joubert C Parra J Hassid S Moulies ME. Echocardiography allows more accurate placement of peripherally inserted central catheters in low birthweight infants. Acta Paediatr. (2013) 102:703–6. 10.1111/apa.12245 23551125

[9] Connolly B Amaral J Walsh S Temple M Chait P Stephens D. Influence of arm movement on central tip location of peripherally inserted central catheters (PICCs). Pediatr Radiol. (2006) 36:845–50. 10.1007/s00247-006-0172-8 16758187

[10] Sharma D Farahbakhsh N Tabatabaii SA. Role of ultrasound for central catheter tip localization in neonates: a review of the current evidence. J Matern Fetal Neonatal Med. (2019) 32:2429–37. 10.1080/14767058.2018.1437135 29397784

[11] Qiong C Yingxin L Yanling H Jun T Zhichun F Dezhi M. Operation and management guidelines for peripherally inserted central catheter in neonates (2021). Zhongguo Dang Dai Er Ke Za Zhi. (2021) 23:201–12. 10.7499/j.issn.1008-8830.2101087 33691911 PMC7969181

[12] Blackwood BP Farrow KN Kim S Hunter CJ. Peripherally inserted central catheters complicated by vascular erosion in neonates. JPEN J Parenter Enteral Nutr. (2016) 40:890–5. 10.1177/0148607115574000 25700180

[13] Gorski LA Hadaway L Hagle ME Broadhurst D Clare S Kleidon T Infusion therapy standards of practice, 8th edition. J Infus Nurs. (2021) 44:S1–S224. 10.1097/NAN.0000000000000396 33394637

[14] Sharpe E Pettit J Ellsbury DL. A national survey of neonatal peripherally inserted central catheter (PICC) practices. Adv Neonatal Care. (2013) 13:55–74. 10.1097/ANC.0b013e318278b907 23360860

[15] Isemann B Sorrels R Akinbi H. Effect of heparin and other factors associated with complications of peripherally inserted central venous catheters in neonates. J Perinatol. (2012) 32:856–60. 10.1038/jp.2011.205 22301530

[16] Telang N Sharma D Pratap OT Kandraju H Murki S. Use of real-time ultrasound for locating tip position in neonates undergoing peripherally inserted central catheter insertion: a pilot study. Indian J Med Res. (2017) 145:373–6. 10.4103/ijmr.IJMR_1542_14 28749401 PMC5555067

[17] Rossi S Jogeesvaran KH Matu E Khan H Grande E Meau-Petit V. Point-of-care ultrasound for neonatal central catheter positioning: impact on x-rays and line tip position accuracy. Eur J Pediatr. (2022) 181:2097–108. 10.1007/s00431-022-04412-z 35152306

[18] Barone G Pittiruti M Biasucci DG Elisei D Iacobone E La Greca A Neo-ECHOTIP: a structured protocol for ultrasound-based tip navigation and tip location during placement of central venous access devices in neonates. J Vasc Access. (2021) 23:679–88. 10.1177/1129729821100770 33818191

[19] Ren XL Li HL Liu J Chen YJ Wang M Qiu RX. Ultrasound to localize the peripherally inserted central catheter tip position in newborn infants. Am J Perinat. (2021) 38:122–5. 10.1055/s-0039-1694760 31412404

[20] Barton A. Confirming PICC tip position during insertion with real-time information. Br J Nurs. (2016) 25(Suppl 2):S17–21. 10.12968/bjon.2016.25.Sup2.S17 27282698

[21] Tawil KA Eldemerdash A Hathlol KA Laimoun BA. Peripherally inserted central venous catheters in newborn infants: malpositioning and spontaneous correction of catheter tips. Am J Perinatol. (2006) 23:37–40. 10.1055/s-2005-921330 16450271

[22] Yan J Zhang XM. A randomized controlled trial of ultrasound-guided pulsed radiofrequency for patients with frozen shoulder. Medicine (Baltimore). (2019) 98:e13917. 10.1097/MD.0000000000013917 30608419 PMC6344187

[23] Mance MJ. Keeping infants warm: challenges of hypothermia. Adv Neonat Care. (2008) 8:6–12. 10.1097/01.ANC.0000311011.33461.a5 18300733

[24] Tander B Baris S Karakaya D Ariturk E Rizalar R Bernay F. Risk factors influencing inadvertent hypothermia in infants and neonates during anesthesia. Paediatr Anaesth. (2005) 15:574–9. 10.1111/j.1460-9592.2005.01504.x 15960641

[25] Mank A van Zanten HA Meyer MP Pauws S Lopriore E Te Pas AB. Hypothermia in preterm infants in the first hours after birth: occurrence, course and risk factors. Plos One. (2016) 11:e0164817. 10.1371/journal.pone.0164817 27812148 PMC5094660

[26] O’Brien EA Colaizy TT Brumbaugh JE Cress GA Johnson KJ Klein JM Body temperatures of very low birth weight infants on admission to a neonatal intensive care unit. J Matern Fetal Neonatal Med. (2019) 32:2763–6. 10.1080/14767058.2018.1446076 29478358 PMC6128769

